# Supplementary material for: Spatial Variation in Stream-Water Bacterial Communities Across Sites Differing in Wild Chinese Giant Salamander Detection Frequency
Source: Biology (Basel). 2026 Sep 18;15(18):1647. doi: 10.3390/biology15181647 (PMC13604818; doi:10.3390/biology15181647)
Supplement: Supplementary file 1 [file biology-15-01647-s001.zip › Data Related to “Spatial Variation in Stream-Water Bacterial Communities across Sites Differing in Wild Chinese Giant Salamander Detection Frequency”/Microbial Data/result/03.TaxonVisual/ternary/sample/phylum/S2-S3-S6.ternary.pdf]

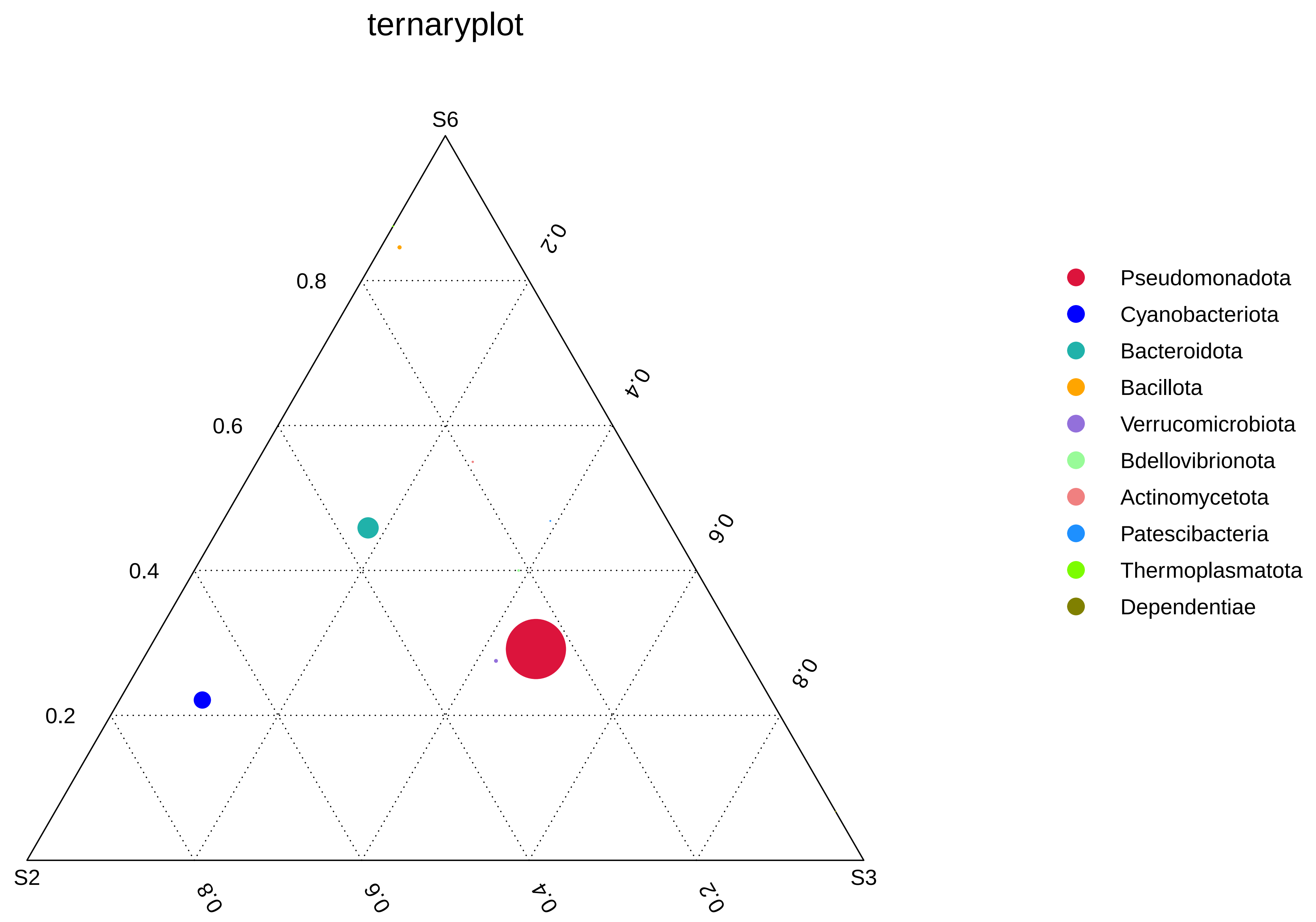
ternaryplot
S6
S2
S3
0.2
0.4
0.6
0.8
Pseudomonadota
Cyanobacteriota
Bacteroidota
Bacillota
Verrucomicrobiota
Bdellovibrionota
Actinomycetota
Patescibacteria
Thermoplasmatota
Dependentiae
